# Supplementary material for: A regression-based method to estimate vessel mass for use in whale-ship strike risk models
Source: PLoS One. 2026 Feb 18;21(2):e0339760. doi: 10.1371/journal.pone.0339760 (PMC12915980; doi:10.1371/journal.pone.0339760)
Supplement: S1 File — (DOCX) [file pone.0339760.s001.docx]

**Results of the linear regression**

**Table S1.** ANOVA table of the linear regression model with the 12 vessel types, with the degrees of freedom (DF), sum square, mean square, F statistics and p value.

| Term | DF | Sum square | Mean square | F value | p |
| --- | --- | --- | --- | --- | --- |
| log(LOA) | 1 | 9400.5 | 9400.5 | 37746.1 | < 0.0001 |
| TYPE | 11 | 268.1 | 24.4 | 97.9 | < 0.0001 |
| log(LOA): TYPE | 11 | 63.3 | 5.8 | 23.1 | < 0.0001 |
| Residuals | 849 | 211.4 | 0.2 |  |  |

**Table S2.** Coefficients of the linear regression, including the estimate, standard error (SE), t value, lower and upper 95% confidence intervals (CI) and p value, with values > 0.05 bolded.

| Vessel type | Intercept | | | | Slope | | | |
| --- | --- | --- | --- | --- | --- | --- | --- | --- |
|  | Estimate  (SE) | t value | 95% CI | p value | Estimate  (SE) | t value | 95% CI | p value |
| Bulk Carrier | 1.729  (1.117) | 1.548 | [-0.463, 3.921] | 0.120 | 3.058  (0.208) | 14.72 | [2.650, 3.466] | **<0.0001** |
| Container Ship | 4.459  (0.917) | 4.862 | [2.659, 6.259] | **<0.0001** | 2.461  (0.168) | 14.63 | [2.131, 2.791] | **<0.0001** |
| Cruise | 4.580  (0.786) | 5.828 | [3.037, 6.122] | **<0.0001** | 2.281  (0.147) | 15.51 | [1.992, 2.569] | **<0.0001** |
| Ferry | 3.225  (0.691) | 4.667 | [1.868, 4.581] | **<0.0001** | 2.619  (0.153) | 17.16 | [2.320, 2.919] | **<0.0001** |
| Fishing | -0.3444  (0.185) | -1.864 | [-0.707, 0.018] | 0.0626 | 3.785  (0.061) | 61.96 | [3.665, 3.905] | **<0.0001** |
| Gov./ Research | 1.083  (0.455) | 2.382 | [0.191, 1.976] | **0.0175** | 3.217  (0.112) | 28.67 | [2.997, 3.437] | **<0.0001** |
| Other | 0.9725  (0.364) | 2.671 | [0.2579, 1.687] | **0.0077** | 3.354  (0.085) | 39.26 | [3.186, 3.522] | **<0.0001** |
| Passenger | 1.463  (0.358) | 4.086 | [0.760, 2.165] | **<0.0001** | 3.081  (0.122) | 25.23 | [2.841, 3.320] | **<0.0001** |
| Pleasure Craft | 3.540  (0.261) | 13.57 | [3.028, 4.052] | **<0.0001** | 2.685  (0.073) | 36.85 | [2.542, 2.828] | **<0.0001** |
| Sailing | 0.2082  (0.298) | 0.6992 | [-0.376, 0.793] | 0.485 | 3.529  (0.106) | 33.21 | [3.321, 3.738] | **<0.0001** |
| Tanker | 1.981  (1.154) | 1.717 | [-0.284, 4.245] | 0.086 | 3.032  (0.219) | 13.85 | [2.602, 3.462] | **<0.0001** |
| Tug | 4.649  (0.508) | 9.154 | [3.652, 5.646] | **<0.0001** | 2.512  (0.136) | 18.45 | [2.245, 2.779] | **<0.0001** |

**Table S3.** Significant differences between the coefficients among all vessel types. The lower-left corner (blue) displays the p-value for the differences in the slope coefficient, and the upper-right corner (green) displays the p-value for the differences in the intercept coefficients.

|  | Bulk Carrier | Container Ship | Cruise | Ferry | Fishing | Gov/Res | Other | Passenger | Pleasure Craft | Sailing | Tanker | Tug |
| --- | --- | --- | --- | --- | --- | --- | --- | --- | --- | --- | --- | --- |
| Bulk Carrier |  | 0.059 | **0.037** | 0.25 | 0.067 | 0.59 | 0.52 | 0.82 | 0.11 | 0.19 | 0.88 | **0.018** |
| Container Ship | **0.026** |  | 0.92 | 0.28 | **< 0.001** | **0.001** | **< 0.001** | **0.0024** | 0.34 | **< 0.001** | 0.093 | 0.86 |
| Cruise | **0.0023** | 0.42 |  | 0.20 | **< 0.001** | **< 0.001** | **< 0.001** | **< 0.001** | 0.21 | **< 0.001** | 0.063 | 0.94 |
| Ferry | 0.089 | 0.49 | 0.11 |  | **< 0.001** | **0.0098** | **0.004** | **0.024** | 0.67 | **< 0.001** | 0.36 | 0.097 |
| Fishing | **< 0.001** | **< 0.001** | **< 0.001** | **< 0.001** |  | **0.0037** | **0.0013** | **< 0.001** | **< 0.001** | 0.12 | **0.047** | **< 0.001** |
| Gov/Res | 0.5 | **< 0.001** | **< 0.001** | **0.0017** | **< 0.001** |  | 0.85 | 0.51 | **< 0.001** | 0.11 | 0.47 | **< 0.001** |
| Other | 0.19 | **< 0.001** | **< 0.001** | **< 0.001** | **< 0.001** | 0.33 |  | 0.34 | **< 0.001** | 0.10 | 0.40 | **< 0.001** |
| Passenger | 0.92 | **0.003** | **< 0.001** | **0.019** | **< 0.001** | 0.41 | 0.067 |  | **< 0.001** | **0.0072** | 0.67 | **< 0.001** |
| Pleasure Craft | 0.09 | 0.22 | **0.014** | 0.7 | **< 0.001** | **< 0.001** | **< 0.001** | **0.0055** |  | **< 0.001** | 0.19 | 0.052 |
| Sailing | **0.044** | **< 0.001** | **< 0.001** | **< 0.001** | **0.037** | **0.044** | 0.2 | **0.0057** | **< 0.001** |  | 0.14 | **< 0.001** |
| Tanker | 0.93 | **0.039** | **0.0045** | 0.12 | **< 0.001** | 0.45 | 0.17 | 0.85 | 0.13 | **0.041** |  | **0.035** |
| Tug | **0.028** | 0.81 | 0.25 | 0.6 | **< 0.001** | **< 0.001** | **< 0.001** | **0.0019** | 0.26 | **< 0.001** | **0.044** |  |

**Figure S1.** Effect of vessel mass (kg) on the probability of lethality of a vessel strike, at seven different transiting speeds (kn), calculated with the biophysical model of Kelley et al. 2021. The default impact area (1.15 m x 1.15 m) and default parameters of an average adult North Atlantic right whale were used.


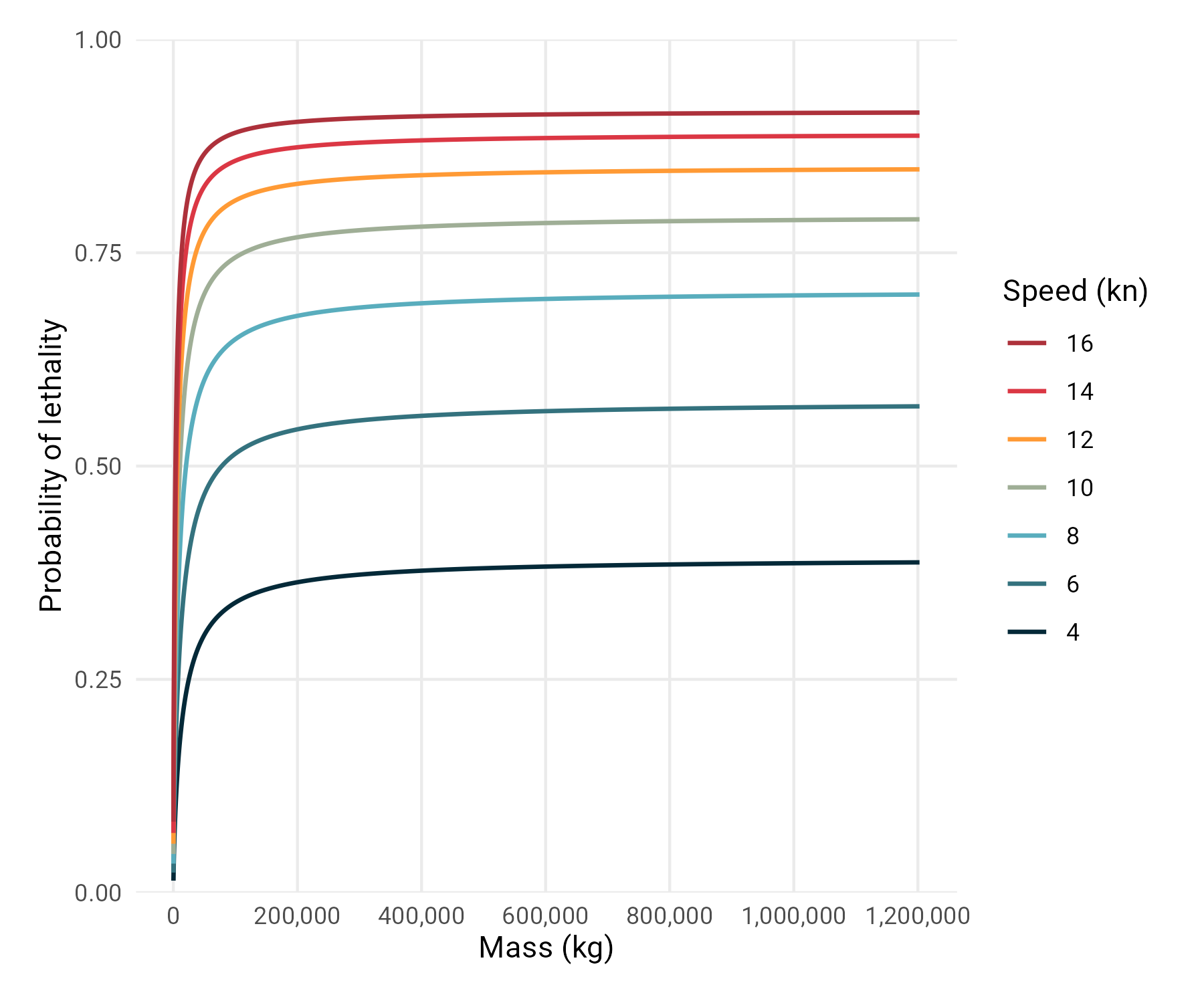


Kelley DE, Vlasic JP, Brillant SW. Assessing the lethality of ship strikes on whales using simple biophysical models. Mar Mammal Sci. 2021 Jan;37(1):251–67. doi:10.1111/mms.12745
