# Supplementary material for: A regression-based method to estimate vessel mass for use in whale-ship strike risk models
Source: PLoS One. 2026 Feb 18;21(2):e0339760. doi: 10.1371/journal.pone.0339760 (PMC12915980; doi:10.1371/journal.pone.0339760)
Supplement: Attachment — Submitted filename: PLOS ONE Review Comments.docx [file pone.0339760.s002.docx]

Summary of paper

The authors present a method to estimate a vessel's total mass (displacement) from its length overall (LOA), a parameter which is routinely available in Automatic Identification System (AIS) datasets. Vessel-strike risk models are increasingly incorporating detailed vessel parameters to estimate whale lethality. However, some of these vessel parameters, such as the mass of a vessel, are often unavailable in AIS data. The authors of this study therefore provide a practical workaround to this limitation by developing vessel-type-specific equations relating LOA to displacement. Using data from over 700 vessels across 11 vessel types, they fit a linear regression model on log-transformed LOA and displacement, finding strong correlations (adjusted R² = 0.954) and significant differences across vessel categories. The resulting equations enable researchers and managers to estimate vessel mass using LOA alone, thereby facilitating broader and more realistic application of strike lethality models that account for vessel size. This addition is useful given that vessel strikes are a significant conservation concern for large whales.

Overall comments

The modelling approach is robust, and the manuscript is clearly written and well-structured. However, the lack of demonstration within a strike lethality model, combined with limited discussion of uncertainty and management application, makes the current version feel methodologically solid but under-utilised. For example, a key limitation of the manuscript is that it does not demonstrate how the estimated vessel mass values would be applied within a strike lethality model, such as Kelley et al. This is particularly problematic given that the title of the paper implies such an application. I would either encourage authors to include at least one worked example showing how some of these vessel mass estimates would be used in a model of vessel strike lethality, such as that of Kelley et al. Doing so would also allow them to validate the applied utility of their method. Or consider editing the title of the manuscript (see my comments below on title).

Line-by-line comments

**P1, L1 (Title)**: The current title implies that the paper directly calculates the lethality of vessel strikes based on vessel characteristics. However, the study does not perform this calculation, nor does it present outputs from a lethality model. I recommend revising the title to accurately reflect the contribution of this manuscript for example, perhaps something along the lines of: “*A regression-based method to estimate vessel mass for use in vessel-strike risk models.*” This highlights the development of a vessel mass estimation method for use in strike lethality modelling, rather than suggesting lethality is being directly calculated here.

**P5, L89**. It would be good to provide the unit of length this measurement is “…(LOA) is the maximum length (in metres?) of a vessel’s…”

**P5, L98**. Perhaps make this sentence a little easier to follow “…as they are useful for docking and maximum cargo allowed onboard” by changing to “…as they provide critical information for docking and guiding the maximum cargo allowed onboard.”

**P6, L106.** In this subheading authors discuss the subset of data used for calculations. I did notice that the table following this first paragraph (Table 1) mentions the online sources, but paragraph of this explanation doesn’t come until later. I would suggest perhaps bringing the last paragraph of this section where non-AIS data is mentioned (L140) to follow on from L120 to make the flow a little easier to follow. And then the paragraph on equations can follow and finishing with “The displacement calculation is described for each of the eleven vessel types in detail below…”.

**P10, L140**. In addition to the comment above, it would be beneficial if the authors could provide more information on how the values from online sources were chosen. The way vessels were chosen could have implications on the results if vessel types were not so dissimilar (and thus dry mass). For example, if all non-AIS data sources were taken from Canadian / US sites, this introduces potential bias, especially if the online-reported vessels are high-end or atypical (e.g., small vessels in the states/Canada may differ to other non-AIS boats like artisanal small vessels in south-east Asia / Africa, or tinny recreational boats from Australia). I guess the point here is, has there been enough effort to capture a range of small non-AIS vessel types around the world, and has there been effort to capture a range of different valued boats (e.g., I am assuming a costly, fancy cat rec boat with modern modifications would differ in mass to same size, plain aluminium boat).

**P9, L137.** I feel like the table subheading is a little ambiguous. I would recommend changing to something like: “Equations used to estimate vessel displacement (Δ) from deadweight tonnage (DWT) in tonnes (1000 kg), based on vessel type and typical vessel length. Equations sourced from Kalokairinos et al. [16], Papanikolaou [13], and IHS Fairplay [17]….”

**P10, L141**. Closing bracket missing after citation.

**P10, L157.** The “Cargo” category aggregates very different vessel types (bulk, container, reefers). Why not maintain subtypes, especially since you use separate equations from Kalokairinos et al.? I would perhaps elaborate more as to why this was done.

**P11, L184.** Is the addition of one average person and luggage the norm for these sorts of calculations? I doubt it makes much difference, but surely if trying to approximate DWT for online sourced vessels, it would not just be the one crew, but the maximum number of passengers the vessel is registered to carry?

**P14, L244.** Could you not refer to your table 2 here instead of the appendix? Same goes to other sections where this is referred to? As a reader, I would not go through Papanikolaou appendix to try and find this equation, but if I am not mistaken you have put that into your table 2? (and referenced it there). So maybe for ease of interpretability by the reader, it would make sense to just refer to your table?

**P16, L279**. What about uncertainty values? Around figure (CI ??) Uncertainty values for the regression….

**P16, L289.** I think authors could expand more on limitations and applicability / generalisability. For example, a brief discussion of how regional variation or temporal evolution in vessel construction might affect the robustness of the LOA–mass relationships when applied more broadly.

**P17, L299**. I feel like this paragraph could be worked on to enhance the impact of this work. For example, this work fills a key data gap that currently limits the operational use of strike lethality models. Its application to AIS datasets has strong potential for management as authors mention. But it can also be integrated into real-time risk assessment and management tools, which could also be mentioned within the manuscript. This could help strengthen the discussion on management applications, which can help elevate the potential value of this work.
