# Supplementary material for: A regression-based method to estimate vessel mass for use in whale-ship strike risk models
Source: PLoS One. 2026 Feb 18;21(2):e0339760. doi: 10.1371/journal.pone.0339760 (PMC12915980; doi:10.1371/journal.pone.0339760)
Supplement: Attachment — Submitted filename: Response to Reviewers.docx [file pone.0339760.s004.docx]

***Reviewer #1***: Yes, the manuscript is technically sound, and the data support the main conclusion that vessel mass can be reliably estimated from LOA and vessel type using a regression-based approach. The statistical analysis is appropriately chosen for the research question. The authors apply a log-log linear regression with vessel type interactions to model the relationship between LOA and displacement, yielding a strong overall fit. However, the manuscript would benefit from greater transparency (e.g., reporting of coefficient uncertainty).

Summary of paper

The authors present a method to estimate a vessel's total mass (displacement) from its length overall (LOA), a parameter which is routinely available in Automatic Identification System (AIS) datasets. Vessel-strike risk models are increasingly incorporating detailed vessel parameters to estimate whale lethality. However, some of these vessel parameters, such as the mass of a vessel, are often unavailable in AIS data. The authors of this study therefore provide a practical workaround to this limitation by developing vessel-type-specific equations relating LOA to displacement. Using data from over 700 vessels across 11 vessel types, they fit a linear regression model on log-transformed LOA and displacement, finding strong correlations (adjusted R² = 0.954) and significant differences across vessel categories. The resulting equations enable researchers and managers to estimate vessel mass using LOA alone, thereby facilitating broader and more realistic application of strike lethality models that account for vessel size. This addition is useful given that vessel strikes are a significant conservation concern for large whales.

Overall comments

The modelling approach is robust, and the manuscript is clearly written and well-structured. However, the lack of demonstration within a strike lethality model, combined with limited discussion of uncertainty and management application, makes the current version feel methodologically solid but under-utilised. For example, a key limitation of the manuscript is that it does not demonstrate how the estimated vessel mass values would be applied within a strike lethality model, such as Kelley et al. This is particularly problematic given that the title of the paper implies such an application. I would either encourage authors to include at least one worked example showing how some of these vessel mass estimates would be used in a model of vessel strike lethality, such as that of Kelley et al. Doing so would also allow them to validate the applied utility of their method. Or consider editing the title of the manuscript (see my comments below on title).

Thank you for taking the time to review our manuscript and provide constructive feedback. Following your recommendations, we have made some changes to the data used and provided more details on the methods and limitations to improve transparency. We have also included an example of the application of the biophysical model and added to the discussion the importance of considering vessel mass and type in ship strike risk assessment and management regulations.

First, based on your comments regarding potential regional bias in the vessel data, we revisited our selected data and changed some of it, particularly that from online sources. We paid closer attention to the origin of the vessels’ builder or manufacturer. We tried to select a range of older and newer vessel models. In some cases, we were unable to find the original advertisement for a boat for sale (archived link), so we replaced it. We also found new sources of information, like the Fishing Vessel Design Database. These useful new resources allowed us to include vessels with a greater variety of designs, compared to primarily North American designs. For the AIS data, we didn’t make any major changes, but we realized that some vessels had replicates of the same model (e.g. a ferry or cruise ship with the exact same LOA and DWT but different ID), which we removed to have a greater variety of different ship models. We also decided not to set a length limit (i.e. including all vessels under a certain length, like we initially did with the cargos and tankers). All of this resulted in slight changes to the dataset, but overall, we hope that it has led to a more randomized and less biased dataset. The results of the regression model did not identify any problems with these changes, and we even improved the accuracy of the model.

Second, we added more detail on data selection, methodology and results. We also added a section in the discussion on limitations and potential variables not accounted for. We included the coefficients and their uncertainty for each equation.

Finally, following your recommendation, we added an example of the calculation of the probability of lethality. Using the equations to convert the LOA to total mass for six different vessel types, we showed how lethality can vary for vessels of the same length. We added some discussion on the implications of accounting for vessel mass and type in risk assessment and management. We referred to current management measures that could be improved by considering more vessel variables.

Overall, these changes did not alter the conclusions of this study, namely that these equations are necessary to use the biophysical model and measure more accurately the probability of lethality of ship strikes. The coefficients may have changed slightly, but with the statistical results, we are confident that the equations reasonably predict the relationship between length and mass.

***Reviewer #2:*** This study provides a fruitful refinement to the biophysical model elaborated by Kelley et al (2021) to estimate the lethality of a collision between a whale and a ship. Such biophysical approach is a potentially more precise way of assessing lethality of a strike than other approaches based on correlational approach as it permits better considering the characteristics of vessels (like size, draught) rather than simply the bessel category (cargo, tanker, sailing, etc). However, the Kelley model is not easy to implement due to the requirements of precise parameters to be known. The present work aimed at facilitating the access to one of these parameters, namely the vessel's displacement (mass). The authors collated the characteristics of a wide array of vessels from several sources and fitted linear régressions to provide user-ready equations converting the vessel length to vessel displacement. The paper is clearly written, the analyses are sound, the Supplementary Files are well constructed and insightful, and I only have a few minor comments to clarify some details in the text, that I detail below.

We thank you for taking the time to review our manuscript. We hope that the changes made help clarify some details in the methods and results.

Line-by-line comments

**L1 (Title)**: The current title implies that the paper directly calculates the lethality of vessel strikes based on vessel characteristics. However, the study does not perform this calculation, nor does it present outputs from a lethality model. I recommend revising the title to accurately reflect the contribution of this manuscript for example, perhaps something along the lines of: “*A regression-based method to estimate vessel mass for use in vessel-strike risk models.*” This highlights the development of a vessel mass estimation method for use in strike lethality modelling, rather than suggesting lethality is being directly calculated here.

This is a good observation and thank you for the suggestion. Although we now added an example of the lethality calculation, we changed the title to better reflect the content of the manuscript.

**Title.** A regression-based method to estimate vessel mass for use in whale-ship strike risk models

**L89:** It would be good to provide the unit of length this measurement is “…(LOA) is the maximum length (in metres?) of a vessel’s…”

That is a good idea. We added the unit in a sentence further on, in addition to the standard unit for DWT.

**L96.** “The LOA and maximum DWT of a vessel are usually available in the static AIS data (in meters and tonnes, respectively), …”

**L98:** Perhaps make this sentence a little easier to follow “…as they are useful for docking and maximum cargo allowed onboard” by changing to “…as they provide critical information for docking and guiding the maximum cargo allowed onboard.”

This is a great suggestion, thank you.

**L98.** “…as they provide critical information for docking and guiding the maximum cargo allowed onboard.”

**L106:** In this subheading authors discuss the subset of data used for calculations. I did notice that the table following this first paragraph (Table 1) mentions the online sources, but paragraph of this explanation doesn’t come until later. I would suggest perhaps bringing the last paragraph of this section where non-AIS data is mentioned (L140) to follow on from L120 to make the flow a little easier to follow. And then the paragraph on equations can follow and finishing with “The displacement calculation is described for each of the eleven vessel types in detail below…”.

We agree, this improves the flow of the text and the information that follows in the tables. We moved the paragraph explaining the online sources right after the description of the AIS sample. (see **L121**)

**L137:** I feel like the table subheading is a little ambiguous. I would recommend changing to something like: “Equations used to estimate vessel displacement (Δ) from deadweight tonnage (DWT) in tonnes (1000 kg), based on vessel type and typical vessel length. Equations sourced from Kalokairinos et al. [16], Papanikolaou [13], and IHS Fairplay [17]….”

We slightly changed your suggestion, but we agree it is now clearer.

**Table 2.** Equations used to estimate vessel displacement (Δ) from deadweight tonnage (DWT) in tonnes (1000 kg), based on each vessel type and typical vessel length overall (LOA) in metres. Equations sourced from Papanikolaou [13] (Table 2.1 and appendixes, referring to Kalokairinos et al. [16] and IHS Fairplay [17]) and expressed to isolate the displacement variable.

**L140:** In addition to the comment above, it would be beneficial if the authors could provide more information on how the values from online sources were chosen. The way vessels were chosen could have implications on the results if vessel types were not so dissimilar (and thus dry mass). For example, if all non-AIS data sources were taken from Canadian / US sites, this introduces potential bias, especially if the online-reported vessels are high-end or atypical (e.g., small vessels in the states/Canada may differ to other non-AIS boats like artisanal small vessels in south-east Asia / Africa, or tinny recreational boats from Australia). I guess the point here is, has there been enough effort to capture a range of small non-AIS vessel types around the world, and has there been effort to capture a range of different valued boats (e.g., I am assuming a costly, fancy cat rec boat with modern modifications would differ in mass to same size, plain aluminium boat).

This is a good point, and we might have been biased toward more North American sources, simply because of the location settings of the search engine and limitations to English websites. When we re-examined our sample and the information on boat dimensions found online, we paid more attention to the origin of the builder or manufacturer. For some of the categories, we modified the sample to try to better reflect the diversity of boat models, focusing less on North America. We also found new sources since the initial search, which helped greatly (e.g. the fishing vessel design database).

For the data coming from boat catalogues and ship brokers, vessels were not restricted to U.S. or Canada. We found vessels designed and sold by companies in Europe, Asia, North America, South America and Oceania. The only region of the world that was noticeably absent was Africa. The sailboat database and the fishing vessel design database captured vessels from all over the world, including designs from Africa, Pacific islands, Caribbeans. Pleasure boats were the most difficult to capture the full range of possibilities because most of the vessels whose displacement or weight data came from company websites, which typically sold new models. Although we had some of these more luxurious models, we also tried to add simpler models (for example, a simple recreational aluminium fishing boat from the Lund Boat Company). It is certainly not perfect but after reviewing the samples and adding new vessel designs, e believe we have a sufficiently large sample that covers a wide variety of models and lengths.

**L141:** Closing bracket missing after citation. **L141**: a bracket is missing after the reference

Corrected.

**L153**: what is the rationale of only using a subset of vessels with LOA > 100 m? (I understand it is to equilibrate the sample size with vessels <100m but it may be good to explicitly state this)

Initially, it was just based on data exploration and trying to capture the range of lengths available in the AIS data. With the separation of the Cargo category into two, we decided to simply randomly sampled 100 vessels. Similarly with the tankers, instead of splitting the sample with a value chosen without specific rationale, we just sampled 100 vessels from the complete AIS data. This way, we might not cover the full range of vessel LOA we had, but the process is more arbitrary.

**L157:** The “Cargo” category aggregates very different vessel types (bulk, container, reefers). Why not maintain subtypes, especially since you use separate equations from Kalokairinos et al.? I would perhaps elaborate more as to why this was done.

The initial idea was to simplify the category in order to reduce the number of equations to deal with. However, upon further examination of the data, we found that there was a significant difference between the equations for bulk carrier and container ship. Although there is a specific equation for reefers, this is not a very common cargo type. In our complete AIS data (over thousands of vessels), there were only 11 reefers. Separating the bulk carrier and the container ship, more common cargo types, make sense since we have distinct equations, and some studies examined them separately. Reefers, however, are not usually examined separately. In the discussion, we added suggestions for dealing with different vessel type categories in a vessel strike risk assessment.

**L386.** “If the vessel type categories presented in this study do not meet user needs for future vessel strike risk assessments, we suggest consulting the appendix to determine which vessel types are significantly different from each other and using only one regression equation in cases where they are not significantly different. Alternatively, if users cannot separate a more general vessel category into different categories (e.g. cargo ships into bulk carriers and container ships, or passenger ships into ferries and cruise ships), we suggest calculating the mass using each equation and averaging the results.”

**L170**: what are Ro-Ro and Ro-Pax?

We added a description of a Ro-Ro and Ro-Pax ferries.

L179. “…for passenger vessels such as ferries, Ro-Ro (wheeled vehicle roll-on/roll-off), and Ro-Pax (roll-on/roll-off with passenger facilities) (Table 2)”.

**L181**: you use data from stern trawlers, yet fishing vessels are in essence quite variable in type, how this may affect the results ?

We did not find a displacement coefficient or equation representing a more general category of fishing vessel. Given that this equation was only used for 39% of the sample and the remainder consisted of vessels whose displacement value was available online and represented a variety of vessel designs, we hope the entire sample captures the variation. We mentioned this limitation in the discussion.

**L328.** “Our classification of vessel types also generalized and aggregated the variety of vessel design and purposes into a few categories. The use of a single equation (e.g. the stern trawler for all larger fishing vessels) may not accurately represent the relationship between LOA and displacement for all vessel in the category.”

**L184:** Is the addition of one average person and luggage the norm for these sorts of calculations? I doubt it makes much difference, but surely if trying to approximate DWT for online sourced vessels, it would not just be the one crew, but the maximum number of passengers the vessel is registered to carry?

The addition of one passenger and one luggage was intended to capture the minimum additional weight that would be included in the total displacement. Since we were taking the time to re-examine each online sample, we followed your suggestion to include the additional weight based on the maximum passenger capacity. Except for two instances where the capacity was not available, we added the weight of one passenger and one luggage, multiplied by the maximum capacity, hopefully better reflecting the maximum displacement, just like the maximum DWT is used for the other equations.

**L129.** “When only the vessel’s net weight was available, which would be the equivalent of the light-ship weight, we estimated the maximum displacement by adding the weight of an average person (75 kg) and one average luggage (20 kg) [13], multiplied by the maximum capacity of the vessel (i.e. maximum number of people onboard).”

**L244:** Could you not refer to your table 2 here instead of the appendix? Same goes to other sections where this is referred to? As a reader, I would not go through Papanikolaou appendix to try and find this equation, but if I am not mistaken you have put that into your table 2? (and referenced it there). So maybe for ease of interpretability by the reader, it would make sense to just refer to your table?

This is a good suggestion for improving readability. We removed the references in the paragraphs (**L158** to **L256**) and instead referred to Table 2, where the references are already described by vessel type.

**L252-261**: what are the response vs predictive variables? what types of models were used, which packages and functions? More details are needed here about the modelling procedure.

We added details about the model structure, including which variables were predictive and which one was the response. The type of model was already described; it is simply a linear regression, using the R function “lm()”. We added the additional packages used for processing data, checking assumptions, and visualizing.

**L258.** “After calculating a displacement value for each vessel and converting the displacement values to kg, we combined data from all vessel type. Based on the equations from Table 2 and after some data exploration, we suspected the relation between the predictive variable LOA and the response variable displacement would follow a power function. In addition, knowing ships are designed and built for specific purposes [16], we expected that different vessel types would have different length-mass relations and therefore, vessel type should be included as a predictive variable. We fit a linear regression on the log transformed ship LOA (log(LOA)) with vessel type as an interaction term to predict the log transformed displacement (log(Δ)).”

**L288.** “All analyses were done in R version 4.5.1 (R Core Team, 2025), using the additional packages “tidyverse” [22] and “ggfortify” [23]. An R code is available in the supporting information.”

**L271-272**: you should mention clearly in the text (probably at the end of the method) how were the equations in Table 3 built: it should be explained how coefficients were retrieved and converted back to natural scale - it would clarify that these equations are indeed user-ready and can be applied without any log-transformation.

We had described how the coefficients were used to build the equations for each vessel type in the R markdown in the supplementary material, but we added these descriptions in the method section of the manuscript as well.

**L268.** “To build more convenient equations describing the displacement from the LOA for each vessel type without the log transformation, we used the model coefficients of the linear function to convert to a power function, where the intercept, b, is transformed to the inverse log, exp(b), and the slope, a, is raised as the power of the predictive variable.”

**L279:** What about uncertainty values? Around figure (CI ??) Uncertainty values for the regression….

The uncertainty values were available in the supplementary material, but we have added them for each equation in Table 3. More details (t-value, 95% CI, p-value) are still available in the Supplementary Material. The 95% CI was already provided around the linear regressions, although it might not be very visible. We added a mention in the figure caption.

**Table 3.** Intercept and slope coefficients from the regression model, with associated standard error (SE). Final equations describing the ship displacement (kg) (Δ) with the length overall (m) (LOA), by vessel type as power functions.

**Fig 3.** Regressions of the log of vessel displacement (kg) by the log of vessel length overall (m), for each vessel type, with the 95% confidence interval shaded in grey. The dark circles represent samples from static AIS data and the orange triangles are samples from online sources (when applicable).

**L289:** I think authors could expand more on limitations and applicability / generalisability. For example, a brief discussion of how regional variation or temporal evolution in vessel construction might affect the robustness of the LOA–mass relationships when applied more broadly.

This is a valid observation. We added details in the discussion on the various limitations of the data and methodology we used in this study, including the regional and temporal variation, as well as the generalization of vessel types. We concluded that, although these variables may impact the regression model, using only two variables in the equations to predict vessel mass keeps the method simple and more accessible to users who may not have access to all the necessary data. With the aim of facilitating the use of the biophysical model and given the overall performance of the model, we are confident that the equations can provide a realistic estimate of mass for use in risk assessments. See paragraphs **L329** and **L342**.

**L299:** I feel like this paragraph could be worked on to enhance the impact of this work. For example, this work fills a key data gap that currently limits the operational use of strike lethality models. Its application to AIS datasets has strong potential for management as authors mention. But it can also be integrated into real-time risk assessment and management tools, which could also be mentioned within the manuscript. This could help strengthen the discussion on management applications, which can help elevate the potential value of this work.

Initially, we did not emphasize applicability too much in order to focus on the method, but we now see the value of including some comments on this subject. With the addition of the example of lethality calculation, we discussed the implications and improvement this could bring to risk assessments. We showed that even if vessels are the same length, they can have different masses due to their function and structure, of the vessel, resulting in different risks of causing lethal injuries to whales. We also refer to the current U.S. management measures, the underestimation of the risk posed by small vessels, and how coastal areas with heavy marine traffic could benefit from a more accurate estimate of risk. See paragraph **L363**.
